# Effects of SiO_2_ Filler in the Shell and Wood Fiber in the Core on the Thermal Expansion of Core–Shell Wood/Polyethylene Composites

**DOI:** 10.3390/polym12112570

**Published:** 2020-11-02

**Authors:** Lichao Sun, Haiyang Zhou, Guanggong Zong, Rongxian Ou, Qi Fan, Junjie Xu, Xiaolong Hao, Qiong Guo

**Affiliations:** 1 Key Laboratory for Bio-based Materials and Energy of Ministry of Education, College of Materials and Energy, South China Agricultural University, 483 Wushan Road, Guangzhou 510642, China; sunlichao@scau.edu.cn (L.S.); zhouhaiyang@scau.edu.cn (H.Z.); rongxian_ou@scau.edu.cn (R.O.); fanqiscience@126.com (Q.F.); junjiexu@stu.scau.edu.cn (J.X.); 2 Key Laboratory of Bio-based Material Science and Technology (Ministry of Education), Northeast Forestry University, Harbin 150040, China; 3 Guangdong Laboratory of Lingnan Modern Agriculture, Guangzhou 510642, China; 4 Art and Design Institute, Yangzhou University, 88 Daxue South Road, Yangzhou 225009, China; ggzong@yzu.edu.cn

**Keywords:** co-extrusion, wood plastic composites, thermal expansion, silica, core–shell structure

## Abstract

The influence of nano-silica (nSiO_2_) and micro-silica (mSiO_2_) in the shell and wood fiber filler in the core on the thermal expansion behavior of co-extruded wood/polyethylene composites (Co-WPCs) was investigated to optimize the thermal expansion resistance. The cut Co-WPCs samples showed anisotropic thermal expansion, and the thermal expansion strain and linear coefficient of thermal expansion (LCTE) decreased by filling the shell layer with rigid silica, especially nSiO_2_. Finite element analysis indicated that the polymer-filled shell was mainly responsible for the thermal expansion. The entire Co-WPCs samples exhibited a lower thermal expansion strain than the cut Co-WPCs samples due to protection by the shell. Increasing the wood fiber content in the core significantly decreased the thermal expansion strain and LCTE of the Co-WPCs. The Co-WPCs whose core layer was filled with 70% wood fiber exhibited the greatest anisotropic thermal expansion.

## 1. Introduction

Wood polymer composites (WPCs) usually consist of moisture-sensitive hydrophilic wood fibers and a temperature-sensitive hydrophobic polymer. The co-extrusion of these two components can prevent moisture absorption by coating a high polymer content in the shell layer [1,2]. In addition, co-extruded wood/polyethylene composites (Co-WPCs) with core–shell structures can also achieve better weatherability [3,4] and fire retardation [5,6] than regular WPCs [1,7]. However, Co-WPCs contain much more polymer in the shell layer, which may increase the thermal expansion more than regular WPCs [8]. Thermal expansion is an important component of dimensional stability, and excessive thermal expansion may restrict the use of Co-WPCs in outdoor applications.

Although some studies have reported that filling rigid materials in the shell layer can decrease the linear coefficient of thermal expansion (LCTE) of the entire Co-WPC, it also introduces some disadvantages, which are summarized in Table 1. The thermal expansion behavior of the Co-WPCs can be greatly affected by the filler loading and type in the shell layer [8]. Adding fibers or spherical particles into the polymer matrix can mechanically restrain polymer chains during heating or cooling cycles, which can decrease the LCTE of composites [9]. The thermal expansion resistance has also been increased by improving the interfacial adhesion between the fillers and matrix [9,10].

Low-cost micro- or nanoscale silica is the most common filler for improving the thermal stability and mechanical properties of polymers [13]. In our previous study, the flexural properties and creep resistance of Co-WPCs were improved by filling the shell layer with nano-silica [14]. In addition, the low LCTE of silica (0.5 ppm/°C) can also decrease the thermal expansion of the resulting composites when added at high concentrations into a polymer matrix (>100 ppm/°C) [13,15]. Therefore, using moderate amounts of silica in the shell may improve the thermal expansion resistance of Co-WPCs.

Similar to silica, the extremely low LCTE of wood fibers (10–30 ppm/°C) can drastically decrease the thermal expansion of polymers. The LCTE of WPCs decreased upon increasing the wood fiber loading [16,17,18]. Less filler in the shell layer, accompanied by high amounts of wood in the core layer, was used to form a high-performance, low-cost Co-WPC [19]. However, solid wood exhibited anisotropic thermal expansion due to the different LCTEs in the longitudinal, radial, and tangential directions [16]. Anisotropic thermal expansion was key to obtaining a tailored single-component LCTE without changing its chemical composition [20]. In addition, the wood fiber orientation in extruded WPCs led to anisotropic mechanical properties, which may have similar effects on the thermal expansion behavior. Thus, the influence of fiber orientation on anisotropic thermal expansion must be considered by filling the fibers with a high aspect ratio [15,21,22].

The objective of this article was to prepare Co-WPCs with silica in the shell layer and wood fiber in the core layer. To optimize the fabrication and thermal expansion resistance, the effects of silica amount in the shell layer and wood fiber in the core layer were investigated for their effects on the anisotropic thermal expansion of Co-WPCs systematically.

## 2. Materials and Methods

### 2.1. Materials

Wood fiber (Populus adenopoda, 40–80 mesh) was prepared using a special crusher. High-density polyethylene (HDPE) pellets (5000 s, 0.95 g cm^−3^) were supplied by the Daqing Petrifaction Company (Daqing, China) with a melt flow rate of 0.90 g 10 min^−1^ according to ASTM 1238. Stearic acid was used as a lubricant and was supplied by Rizhisheng Company (Nantong, China) with a melting point of 65 °C and a density of 0.85 g cm^−3^. Maleic anhydride-grafted polyethylene (MAPE) compatibilizer was supplied by Rizhisheng Company (Nantong, China) with a melt flow rate of 1.7 g 10 min^−1^. Two forms of silica were used: microscale silica (mSiO_2_) with an average diameter of approximately 5 µm (Xiang Lan Chemical Co., Ltd., Shanghai, China) and nanoscale silica (nSiO_2_) with an average diameter of 15 nm (Shanghai Meng Tai Hu Industrial Co., Ltd., Shanghai, China) [14].

### 2.2. Preparation of the Composites

The wood fiber was dried in a drying oven for 24 h at 103 ± 0.1 °C before being melt blended with HDPE, compatibilizer, and lubricant using a twin-screw extruder (L/D ratio of 30, SJSH-30, Nanjing Rubber Machinery Factory, Nanjing, China) to prepare WPC pellets for the core (Table 2). The temperatures ranged from 145 to 165 °C. The nSiO_2_ or mSiO_2_ particles were initially melt blended with HDPE at the same temperature range using a twin-screw extruder in a specific ratio (Table 2). The resulting blends of HDPE, silica, and wood fiber were used as the shell layer using the same processing parameters. The shell layer granules were hot-pressed into a 4 mm layer at 180 °C with a pressure of 10 MPa for thermal expansion measurements.

The core and shell layer granules were extruded using co-extrusion equipment to prepare Co-WPCs, which the co- extrusion equipment including a single-screw extruder with L/D ratio of 45 (SJ-45, Nanjing Rubber Machinery Factory, Nanjing, China) and 30 (SJ-30, Nanjing SKY WIN Sci. & Tech. Dev. Co., Ltd., Nanjing, China), respectively. The resulting Co-WPCs were square, with sizes of 45 × 6 mm^2^ (length × width) and a 1 mm shell thickness. The core layer without a shell layer (45 × 6 mm^2^) was used as the control.

### 2.3. Thermomechanical Analysis (TMA)

**Figure 1:**
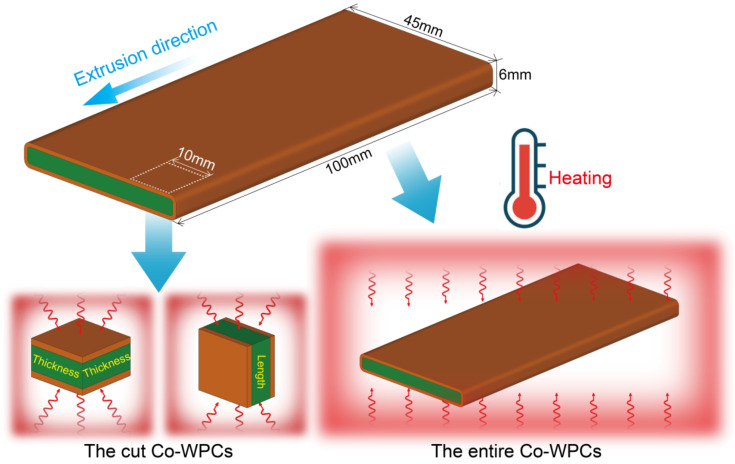
Schematic of the cut and entire Co-WPCs samples for thermal expansion tests.

The thermal expansion of the cut Co-WPCs samples (10 × 10 × 6 mm^3^) was tested by a Q400 thermomechanical analyzer (TA Instruments Inc., New Castle, DE, USA). Before testing, all samples were heated at 60 °C for 24 h to eliminate the thermal history. Tests were run from −30 to 90 °C under a high-purity nitrogen atmosphere with a 50 mL min^−1^ flow rate and a heating rate of 3 °C min^−1^. The thermal expansion along the thickness and extrusion direction (length) was measured (Figure 1).

The thermal expansion measured using TMA was unsuitable for the Co-WPCs samples due to their large sizes. The entire Co-WPC samples (100 × 45 × 6 mm^3^) were first measured at their original sizes at 25 °C, and then their expanded sizes were recorded after being heated in an oven at 60 °C for 24 h to calculate the thermal expansion ratio. The thermal expansion of the entire Co-WPC samples along the thickness and extrusion direction (length) was measured (Figure 1).

### 2.4. Morphological Analysis

Thin sections with a thickness of 0.10–0.13 mm were cut from the Co-WPC profiles along and cross-planar transverse to the extrusion direction, respectively. The core–shell interface and wood fiber orientation were measured by a SMART-POL optical microscope (Chongqing Optec Instrument Co., Ltd., Chongqing, China).

### 2.5. Finite Element Analysis (FEA)

For simplicity, both the core and shell layers of the Co-WPCs were assumed to be isotropic. Abaqus 6.13 FEA software was used to numerically analyze the thermal expansion of the Co-WPCs samples using the parameters shown in Table 3. All sample dimensions used in the geometrical model were the same as those used during thermal expansion tests.

## 3. Results

### 3.1. Subsection

**Figure 2:**
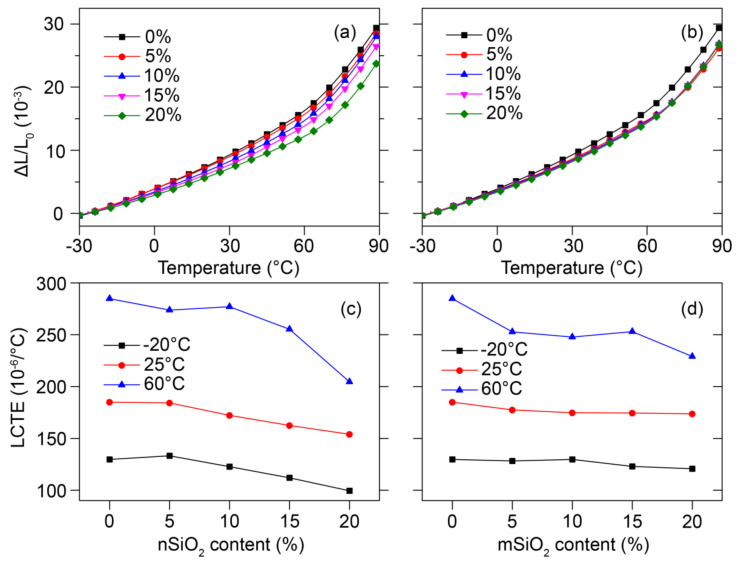
Thermal expansion strain of the shell layer samples: (**a**) nSiO_2_ and (**b**) mSiO_2_ fillers in shell layer, and LCTE as a function of silica content: (**c**) nSiO_2_ and (**d**) mSiO_2_ fillers in the shell layer.

The thermal expansion strain of the shell layer without filler (S0) was 29.74 ‰ at 90 °C and decreased to 18.96% for nSiO_2_ (S20) and 8.84% for mSiO_2_ (S20) (Figure 2a,b). This indicates that the thermal expansion resistance of the shell was greatly improved by adding nSiO_2_, which has been shown to improve the mechanical properties by forming immobilization sites on HDPE chains via van der Waals forces [14,24,25]. These immobilization sites physically and mechanically restrained the HDPE matrix and improved the thermal resistance of the composites [13]. However, adding mSiO_2_ only moderately and nonlinearly reduced the thermal expansion strain (Figure 2b), suggesting that mSiO_2_ only had a small effect on the polymer matrix [26,27,28]. The reason can be explained by noting that the number of SiO_2_ particles per unit volume increased 10^9^ times when changing from micro- to nanoscale SiO_2_, which led to a significantly higher interfacial area between the SiO_2_ and the polymer matrix [13].

The LCTE was obtained from the slope of the linear portion of the thermal expansion strain curve (Figure 2c,d). The LCTE of all samples increased upon increasing the temperature from −20 to 60 °C, illustrating the temperature sensitivity of the polymer LCTE. However, the LCTE significantly decreased upon increasing the nSiO_2_ content, especially at 60 °C (≈60% decrease). Similar to the thermal expansion strain, the LCTE only moderately decreased after the addition of mSiO_2_, indicating that the LCTE of the WPCs depended mainly upon the polymer matrix [17]. In addition, using low-LCTE rigid fillers may reduce the thermal expansion of the resulting polymer composites [9,13,15,29].

### 3.2. Thermal Expansion Anisotropy of Co-WPCs Silica Filler in the Shell Layer

**Figure 3:**
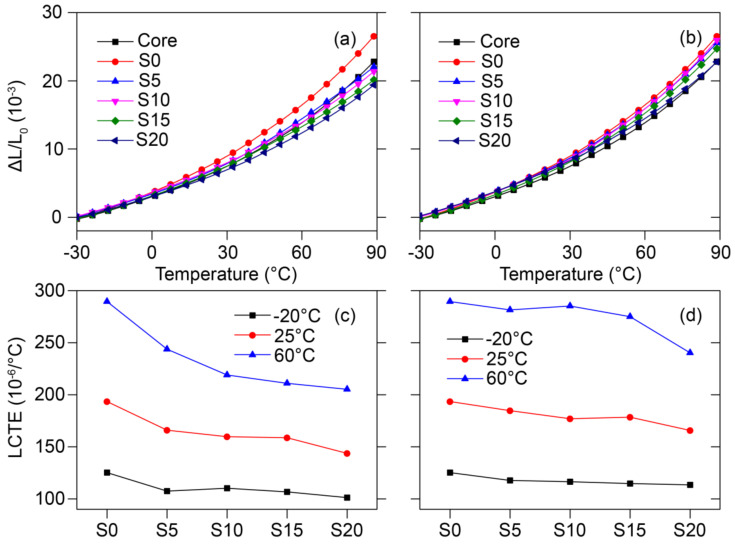
Thermal expansion strain of the cut Co-WPCs in the thickness direction: (**a**) nSiO_2_ and (**b**) mSiO_2_ fillers in shell layer, and LCTE as a function of silica content: (**c**) nSiO_2_ and (**d**) mSiO_2_ fillers in the shell layer.

The co-extruded sample (S0) exhibited a 16.41% higher thermal expansion strain at 90 °C than the pure core layer in the thickness direction (Figure 3a). This was attributed to the small amount of wood fiber in the shell layer, which had only a slight stiffness. Incorporating nSiO_2_ in the shell considerably decreased the thermal expansion strain of Co-WPCs because of its smaller size effect, which was lower than the core layer, and S20 showed the largest decrease. Adding mSiO_2_ in the shell only slightly decreased the thermal expansion strain compared with nSiO_2_, but it was still higher than the pure core layer (Figure 3b). The lack of a positive effect of mSiO_2_ on the LCTE can be viewed as the absence of nanoscale effects, which is consistent with the shell layer results. Since the enhanced mechanical properties of the shell contributed to those of the entire Co-WPC [7,8], these results illustrate that incorporating rigid SiO_2_ in the shell layer can substantially improve the thermal expansion resistance of the Co-WPCs.

**Figure 4:**
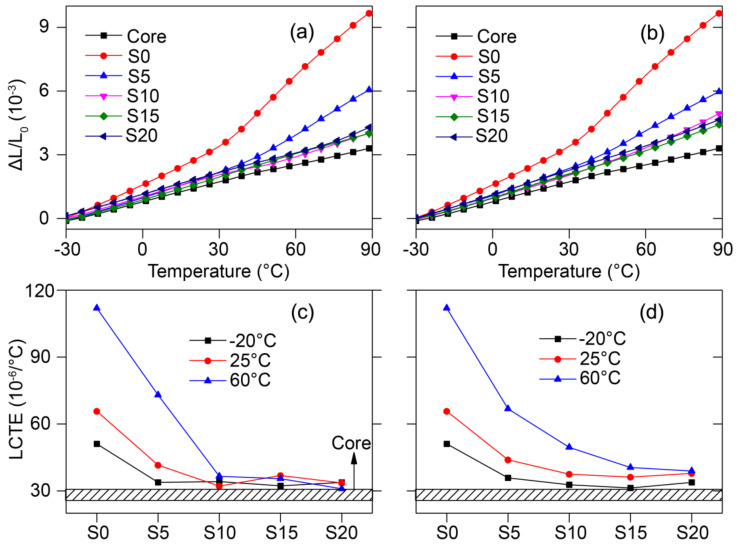
Thermal expansion strain of the cut Co-WPCs in the extrusion direction: (**a**) nSiO_2_ and (**b**) mSiO_2_ fillers in shell layer, and LCTE as a function of silica content: (**c**) nSiO_2_ and (**d**) mSiO_2_ fillers in the shell layer.

**Figure 5:**
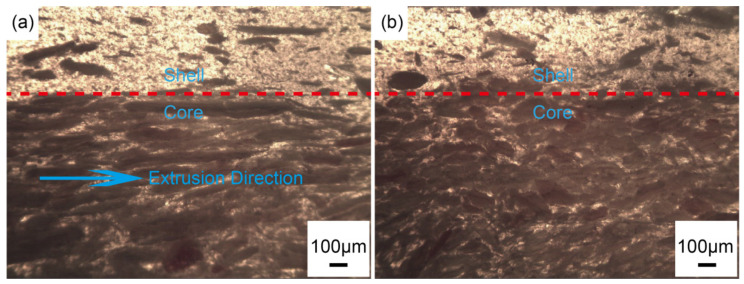
Optical micrographs of Co-WPCs (S0) along the extrusion direction ((**a**) ×40) and in the cross-section transverse to the extrusion direction ((**b**) ×40).

The Co-WPCs exhibited much lower thermal expansion strain in the length (or extrusion) direction than along the thickness direction (Figure 4), which may be due to the orientation effect of wood fiber, especially in the core. On the sectioned surface of the Co-WPCs, wood fibers were oriented in the shell and core layers along the extrusion direction due to the high extrusion pressure (Figure 5a) [30,31]. In addition, the shell and core layers exhibited proper interfacial adhesion, since the same HDPE matrix was used to integrate the two layers into a coherent material at sufficient temperature and pressure. However, the wood fibers were randomly distributed in the cross-section transverse to the extrusion direction (Figure 5b), which lead to a higher thermal expansion strain in the thickness direction. In the extrusion direction, high-aspect-ratio wood fiber decreased the LCTE of the WPCs to mechanically restrain the polymer matrix from deforming by changing the thermal stress distribution inside the WPCs [13]. The high-aspect-ratio fillers restricted the polymer chain relaxation more than the spherical fillers [13]. In addition, the thermal expansion strains of Co-WPCs decreased remarkably by filling the shell with nSiO_2_ or mSiO_2_ compared with S0; however, they were still higher than the core layer (Figure 4a,b). The LCTE values greatly decreased upon increasing the nSiO_2_ content and exhibited comparable levels to the core layer in S10, even when heated from −20 to 60 °C (Figure 4c). When mSiO_2_ was incorporated in the shell layer, the LCTE only slightly increased when the shell layer changed from S10 to S20 (Figure 4d). These results suggest that the thermal expansion was dominated by the oriented core layer, and the rigid SiO_2_ filler in the shell produced a cooperative effect that decreased thermal expansion in the extrusion direction. However, the LCTE in the extrusion direction was lower in the thickness direction, which may lead to greater thermal expansion when the dimensions are much larger in the extrusion direction in practical applications.

**Figure 6:**
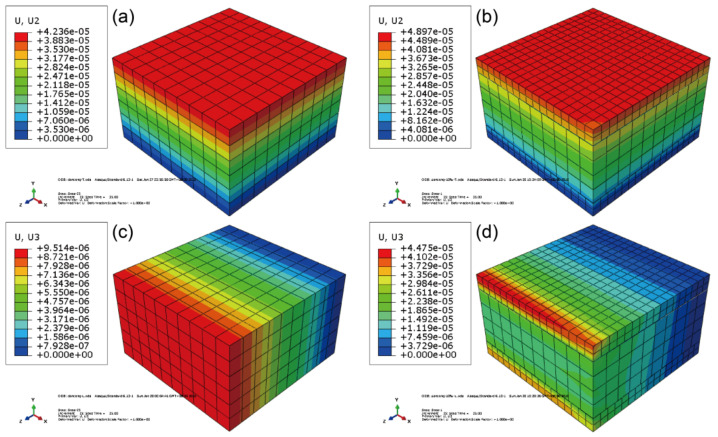
Simulated thermal expansion strain of the cut Co-WPCs in the thickness direction: (**a**) single core layer and (**b**) Co-WPCs (S0); in the extrusion directions: (**c**) single core layer and (**d**) Co-WPCs (S0).

To analyze why anisotropic thermal expansion occurred in the Co-WPCs, the thermal expansion behavior was simulated by FEA from 25 to 60 °C in the thickness and extrusion directions (Figure 6). For brevity, the core and shell layers of Co-WPCs were assumed to be homogenous and only showed different LCTE values in the thickness (y-axis) and extrusion (z-axis) directions. Higher thermal expansion values were observed for Co-WPCs than the control, indicating poorer thermal expansion resistance. The shell layer was the main body that underwent thermal expansion in the extrusion direction due to its higher LCTE, showing that the wood fiber orientation determined the anisotropic expansion of the Co-WPCs.

**Figure 7:**
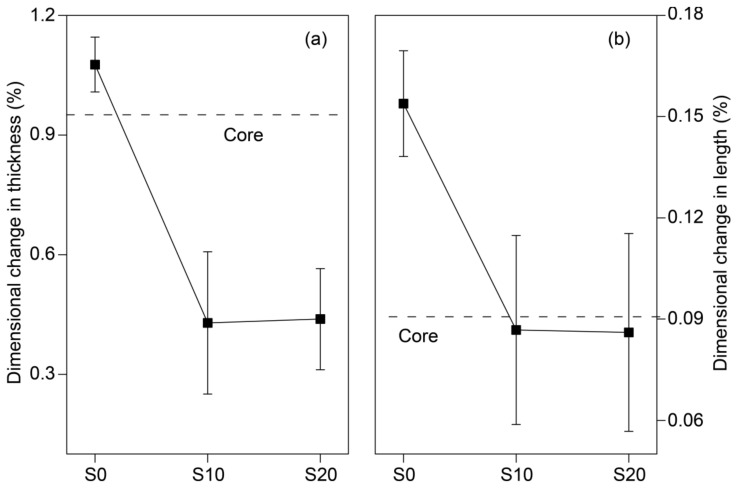
Thermal expansion strain of the entire Co-WPCs with nSiO_2_ filler in the shell in the thickness (**a**) and length directions (**b**).

The thermal expansion specimens used for TMA were cut from Co-WPCs, which cannot reflect the protective effect of the coating shell layer, and the thermal expansion behavior of the entire Co-WPCs samples (100 mm × 45 mm × 6 mm) was further analyzed. The thermal expansion strain showed the same magnitude as the above results but was much lower (Figure 7), indicating that the coating shell layer can mechanically restrict the entire Co-WPCs. After using rigid silica filler in the shell, the entire Co-WPCs (S10 and S20) showed smaller dimensional changes than the core layer (control) in the thickness and extrusion directions. The simulation results also demonstrated the protective effect of the coating shell layer on the thermal expansion resistance of the entire Co-WPCs (Figures S1 and S2).

### 3.3. Thermal Expansion Anisotropy of Co-WPCs with High Filler Contents in the Core

Increasing the wood fiber from 10% to 30% linearly decreased the thermal expansion strain of the shell layer (Figure S3). The thermal expansion strain and LCTE of Co-WPCs were also moderately reduced by increasing the wood fiber in the shell layer but were still higher than those of the control (Figure S4), which demonstrates a lower efficiency than rigid silica. Upon increasing the amount of wood fiber in the shell layer, the entire Co-WPCs showed a smaller dimensional change in the thickness direction and comparable dimensional change in extrusion direction than the control (Figure S5).

**Figure 8:**
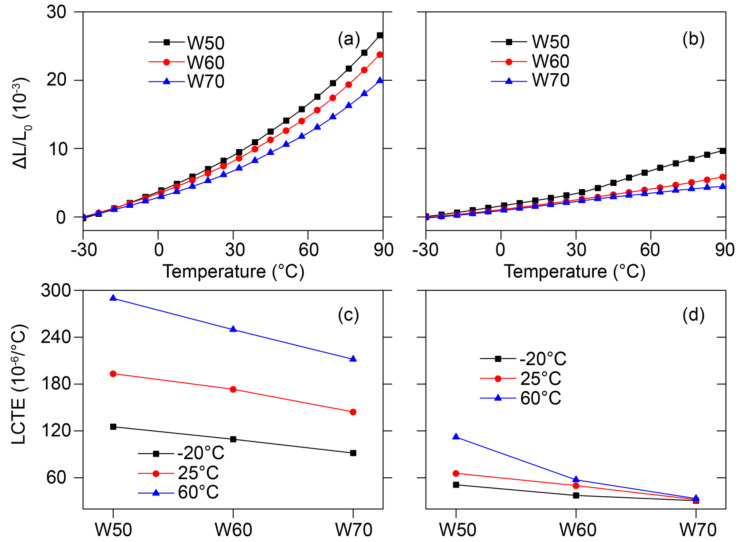
Thermal expansion strain as a function of temperature of Co-WPCs with high filler content: (**a**) in the thickness and (**b**) length directions, and LCTE in the thickness (**c**) and length directions (**d**).

Therefore, the effect of high wood fiber filler contents in the core layer on the anisotropic thermal expansion of Co-WPCs was analyzed (Figure 8). Increasing the wood fiber to 70% in the core layer distinctly decreased the thermal expansion strain of Co-WPCs in the thickness and extrusion directions (Figure 8a,b). The LCTE values also linearly decreased, especially at 60 °C (Figure 8c,d), which meant that the wood fiber ratio of the core primarily determined the thermal expansion of Co-WPCs. Core layers with high filler contents also exhibited greater anisotropic thermal expansion due to the lower LCTE in the extrusion direction.

## 4. Conclusions

The thermal expansion strain of cut Co-WPCs samples decreased upon increasing the silica content in the shell layer, especially for nSiO_2_. The LCTE values of Co-WPCs also decreased upon increasing the silica content but showed anisotropic thermal expansion due to the wood fiber orientation in the core layer. Increasing the rigid silica content in the shell was an effective method to reduce the thermal expansion strain of the Co-WPCs. The entire Co-WPCs samples exhibited lower thermal expansion strain than the cut Co-WPCs samples, illustrating the protective effect of the coating shell layer. The high wood fiber filler content in the core decreased the thermal expansion strain and LCTE in the thickness and extrusion directions, respectively. Thus, the optimized formulation on both the shell and core layers is a suitable method for optimizing the thermal expansion resistance of Co-WPCs.

## Figures and Tables

**Table 1:** Summary of combinations of core–shell layer fillers and their effects on the thermal expansion of co-extruded wood/polyethylene composites (Co-WPCs).

Core Formulation	Shell Formulation	Variables	LCTE	Demerits	Refs
WF:HDPE:MAPE:Lubricant: = 50:40:4:6	HDPE/GF	Shell GF (0–40%)	↓	Only exceeded GF content (>30%) decreased LCTE	[11]
HDPE:WF:Lubricant:MAPE = 40:50:6:4	HDPE/WF/TPCC	Shell WF (0–25%), TPCC (6–18%)	↑	Only weak core lead to decreased LCTE	[8]
WF:HDPE:Talc:Lubricant:MAPE = 55:33:5:5:2	HDPE/BF	Shell BF (0–30%)	↑	LCTE of shell decreased, but increased LCTE of Co-WPCs	[9]
WF:HDPE:Talc:Lubricant:MAPE = 55:33:5:5:2	HDPE/Talc	Shell Talc (0–50%)	↑	LCTE of shell decreased, but increased LCTE of Co-WPCs	[10]
WF:HDPE:Talc:Lubricant:MAPE = 55:33:5:5:2	HDPE/BF/Talc	BF/Talc = 0/30, 10/20, 15/15, 20/10,30/0 wt % in shell	↑	LCTE of shell decreased slightly, but without LCTE of Co-WPCs	[12]

*Note*: WF = Wood fiber, HDPE = High-density polyethylene, MAPE = Maleic anhydride-grafted polyethylene, GF = Glass fiber, BF = Basalt fiber, TPCC = Treated precipitated calcium carbonate, LCTE = Linear coefficient of thermal expansion, ↑ = Increase, and ↓ = Decrease.

**Table 2:** Formulations of Co-WPCs filled with various contents of micro- or nanoscale silica and wood in the shell and core layers.

Sample ^1^	Shell Layer (wt %)			Core Layer (wt %)			
	WF	HDPE	Silica	WF	HDPE	MAPE	Lubricant
Core	0	0	0	50	45	3	2
S0	10	90	0	50	45	3	2
S5	10	90	5	50	45	3	2
S10	10	90	10	50	45	3	2
S15	10	90	15	50	45	3	2
S20	10	90	20	50	45	3	2
W50	10	90	0	50	45	3	2
W60	10	90	0	60	35	3	2
W70	10	90	0	70	25	3	2

^1^ W and S represent the wood fiber and silica, respectively, and the number behind W and S indicates the weight content.

**Table 3:** The measured parameters of wood polymer composite (WPC) core and shell layers at 25 °C were used for finite element analysis [18,23].

Type	Young’s Modulus (GPa)	Poisson Ratio	Density (g cm^−3^)	Average LCTE (25→60 °C) (10^−6^ °C^−1^)
Core Layer	2.0	0.30	1.2	209 ^a^/33 ^b^
S0 Shell Layer	0.71	0.38	0.95	234

^a^ and ^b^ represent the average LCTE of the core layer in thickness and extrusion direction, respectively.

## Supplementary Materials

The supplementary materials are available online at https://www.mdpi.com/2073-4360/12/11/2570/s1, Figure S1: Simulated thermal expansion strain values the entire Co-WPCs in the thickness direction: (a) single core layer (W50), (b) S0, (c) S10 and (d) S20, Figure S2: Simulated thermal expansion strain values of the entire Co-WPCs in the extrusion direction: (a) single core layer (W50), (b) S0, (c) S10 and (d) S20, Figure S3: Thermal expansion strain of the shell filling with different wood fiber contents, Figure S4: Thermal expansion strain of the cut Co-WPCs (W50) filling with different wood fiber in shell layer in the thickness (a) and extrusion directions (b); and LCTE in thickness direction (c) and extrusion directions (d), Figure S5: Thermal expansion strain of the entire Co-WPCs (W50) filling with different wood fiber in shell layer in the thickness (a) and extrusion directions (b).
