# Supplementary material for: Effects of SiO2 Filler in the Shell and Wood Fiber in the Core on the Thermal Expansion of Core–Shell Wood/Polyethylene Composites
Source: Polymers (Basel). 2020 Nov 2;12(11):2570. doi: 10.3390/polym12112570 (PMC7692157; doi:10.3390/polym12112570)
Supplement: Supplementary file 1 [file polymers-12-02570-s001.pdf]

Effects of SiO_2 filler in the shell and wood fiber in the core on the thermal expansion of core-shell wood/polyethylene composites

Lichao Sun [a, b, c, 1], Haiyang Zhou [a, c, 1], Guanggong Zong [d], Rongxian Ou [a, c], Qi Fan [a, c], Junjie Xu [a, c], Xiaolong Hao [a, c, *], Qiong Guo [a, c, *]

[a] Key Laboratory for Biobased Materials and Energy of Ministry of Education, College of Materials and Energy, South China Agricultural University, 483 Wushan Road, Guangzhou 510642, P. R. China

[b] Key Laboratory of Bio-ased Material Science and Technology (Ministry of Education), Northeast Forestry University, Harbin, Heilongjiang, 150040, China

[c] Guangdong Laboratory of Lingnan Modern Agriculture, Guangzhou, 510642, China

[d] Art and Design Institute, Yangzhou University, 88 Daxue South Road, Yangzhou 225009, P. R. China

[1] These authors contributed equally to this work.

* Correspondence to: Xiaolong Hao, haoxiaolong@scau.edu.cn; Qiong Guo, email: guoqiong@scau.edu.cn

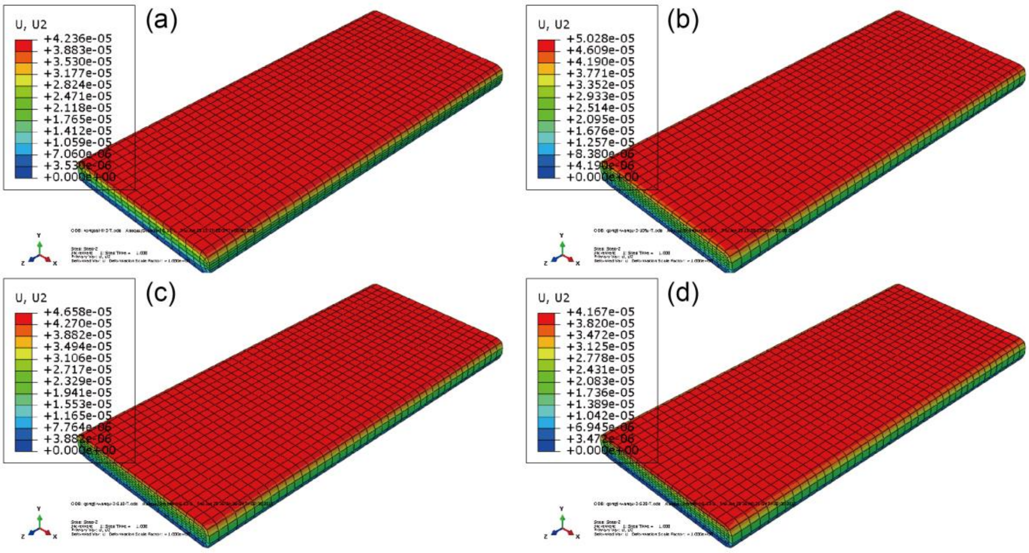

FS. 1 Simulated thermal expansion strain values the entire Co-WPCs in the thickness direction: (a) single core layer (W50), (b) S0, (c) S10 and (d) S20.

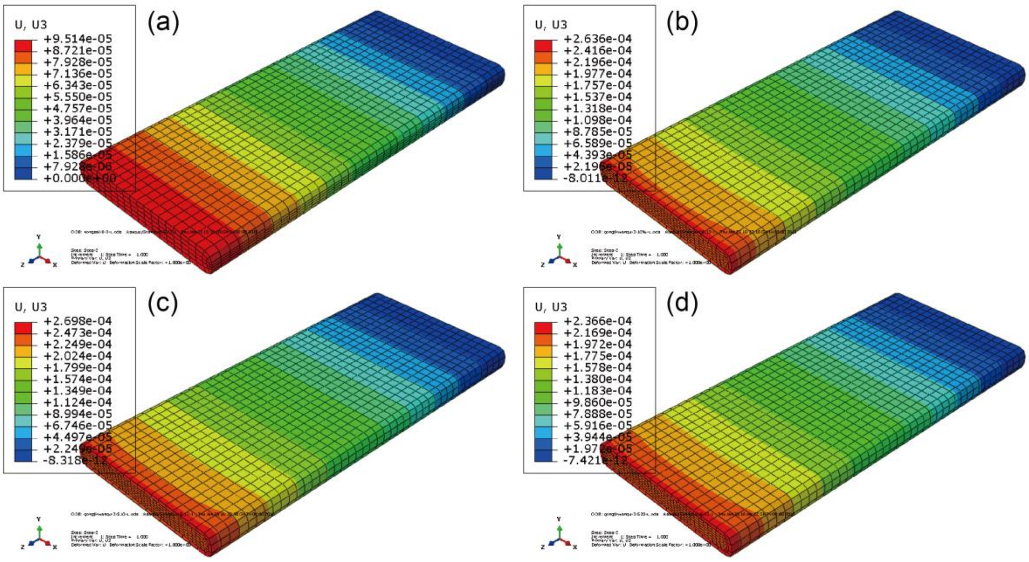

FS. 2 Simulated thermal expansion strain values of the entire Co-WPCs in the extrusion direction: (a) single core layer (W50), (b) S0, (c) S10 and (d) S20.

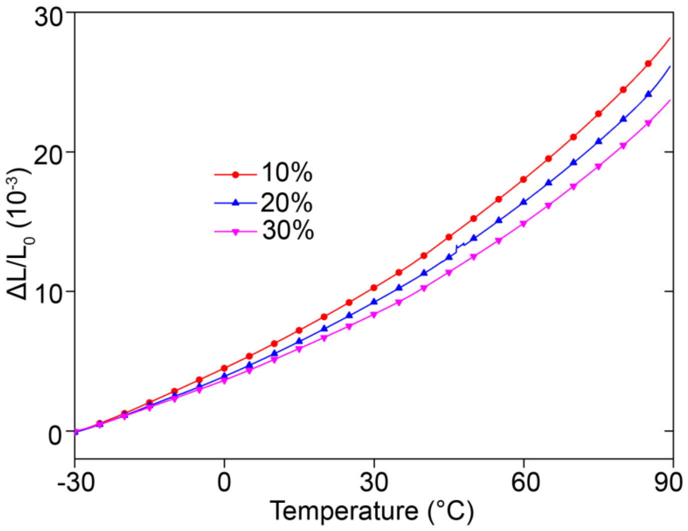

FS. 3 Thermal expansion strain of the shell filling with different wood fiber contents.

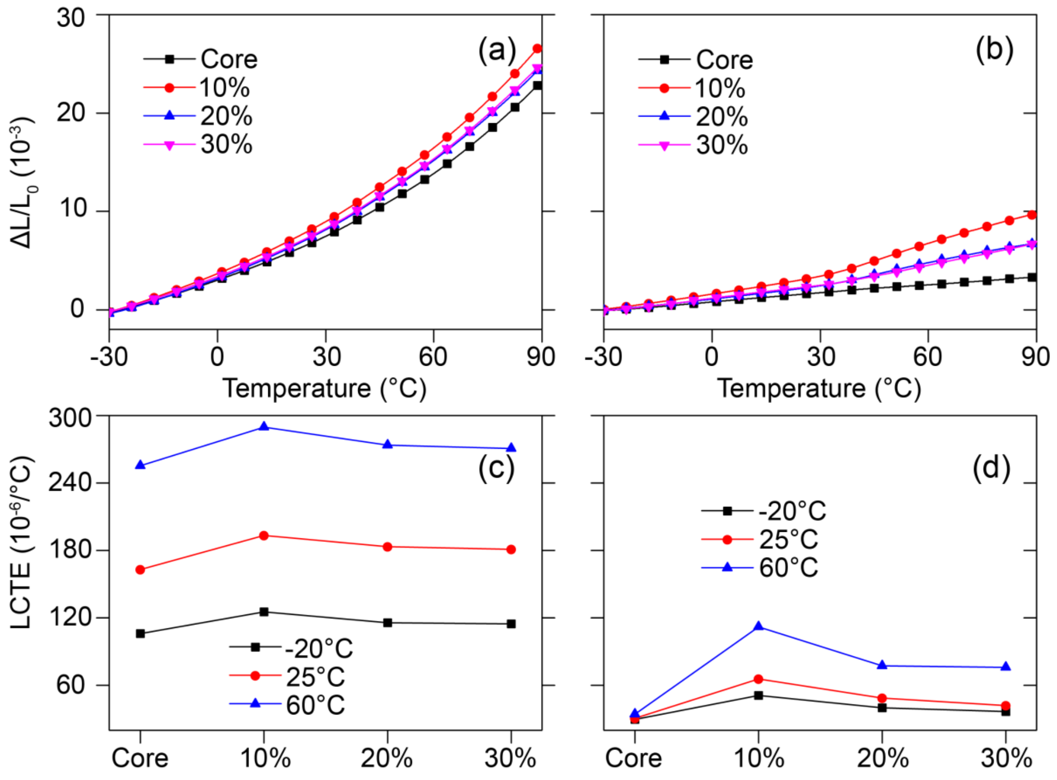

FS. 4 Thermal expansion strain of the cut Co-WPCs (W50) filling with different wood fiber in shell layer in the thickness (a) and extrusion directions (b); and LCTE in thickness direction (c) and extrusion directions (d).

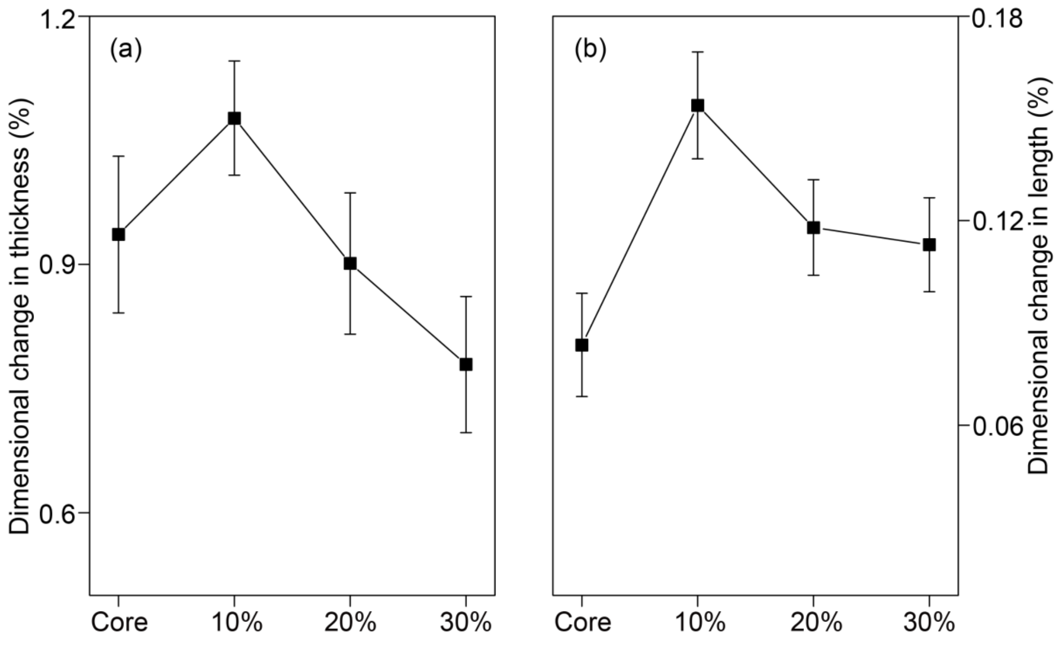

FS. 5 Thermal expansion strain of the entire Co-WPCs (W50) filling with different wood fiber in shell layer in the thickness (a) and extrusion directions (b).
